# Predicting restoration of kidney function during CRRT-free intervals

**DOI:** 10.1186/1749-8090-7-6

**Published:** 2012-01-18

**Authors:** Daniel Heise, Daniel Gries, Onnen Moerer, Annalen Bleckmann, Michael Quintel

**Affiliations:** 1 Department of Anesthesiology, Emergency and Critical Care Medicine, University Hospital Göttingen, Germany; 2 Department of Anaesthesiology and Critical Care Medicine, Emergency Medicine and Pain Therapy, Hospital Dresden-Friedrichstadt, Dresden, Germany; 3 Department of Medical Statistics, University Hospital Göttingen, Germany

## Abstract

**Background:**

Renal failure is common in critically ill patients and frequently requires continuous renal replacement therapy (CRRT). CRRT is discontinued at regular intervals for routine changes of the disposable equipment or for replacing clogged filter membrane assemblies. The present study was conducted to determine if the necessity to continue CRRT could be predicted during the CRRT-free period.

**Materials and methods:**

In the period from 2003 to 2006, 605 patients were treated with CRRT in our ICU. A total of 222 patients with 448 CRRT-free intervals had complete data sets and were used for analysis. Of the total CRRT-free periods, 225 served as an evaluation group. Twenty-nine parameters with an assumed influence on kidney function were analyzed with regard to their potential to predict the restoration of kidney function during the CRRT-free interval. Using univariate analysis and logistic regression, a prospective index was developed and validated in the remaining 223 CRRT-free periods to establish its prognostic strength.

**Results:**

Only three parameters showed an independent influence on the restoration of kidney function during CRRT-free intervals: the number of previous CRRT cycles (medians in the two outcome groups: 1 vs. 2), the "Sequential Organ Failure Assessment"-score (means in the two outcome groups: 8.3 vs. 9.2) and urinary output after the cessation of CRRT (medians in two outcome groups: 66 ml/h vs. 10 ml/h). The prognostic index, which was calculated from these three variables, showed a satisfactory potential to predict the kidney function during the CRRT-free intervals; Receiver operating characteristic (ROC) analysis revealed an area under the curve of 0.798.

**Conclusion:**

Restoration of kidney function during CRRT-free periods can be predicted with an index calculated from three variables. Prospective trials in other hospitals must clarify whether our results are generally transferable to other patient populations.

## Introduction

Acute impairment of kidney function is common in critically ill patients. Although the individual risk varies widely depending on the underlying disease, the overall incidence is 15-20% [1,2]. Secondary complications such as hypervolemia or electrolyte disturbances can be effectively treated by renal replacement therapy. Although their superiority to intermittent therapies is not yet proven, continuous renal replacement therapies (CRRT) are used predominantly in critically ill patients, because the gradual removal of fluids is tolerated better, especially in hemodynamically instable patients [3,4]. However, even continuous treatments must be regularly interrupted because the maximum operation time of disposable products is usually limited to 72 hours. Moreover, blood clots in the filter cartridge and increasing flow resistance in the venous catheters can also require unscheduled cessation of therapy.

Most patients require several CRRT cycles but renal function recovers in the vast majority of cases [5]. If there are no mandatory indications for immediate continuation of CRRT (e.g. severe hyperkalemia) during a CRRT-free interval, the attending physician must carefully assess whether a further treatment cycle is necessary or not. In addition to medical considerations, this decision also affects the utilization of resources because setting up hemofiltration devices requires significant expenditures with regard to personnel and material. At present, clinicians practice CRRT in very different ways [6], and there are only a few evidence-based recommendations on how CRRT should be performed [7], and at which point CRRT should be started or discontinued [8]. To our knowledge, only two studies on predicting the recovery of renal function during CRRT-free intervals have been published [9,10]. Therefore, the aim of the present study was to evaluate whether the need for a further CRRT cycle can be determined on the basis of suitable parameters after cessation of a CRRT-cycle.

## Materials and methods

### Patients

In the period from 2003 to 2006, 7471 patients were treated on our surgical ICU, of whom 605 required CRRT. The only exclusion criterion was pre-existing end-stage renal failure requiring dialysis. Complete data sets for all CRRT-free intervals were available for 222 patients. There was a total of 448 CCRT-free intervals, which were used for analysis.

### Criteria for terminating and resuming CRRT

According to the standard operating procedures of our ICU, the following two rules were strictly binding for the decision to stop or restart CRRT:

• Every CRRT cycle is continued until either the filter is obstructed by clots or the maximum operating time of the disposable CRRT material is reached, at which time CRRT is stopped and the device disassembled. This rule is also adhered to in patients with increasing urinary output during CRRT, because glomerular filtration is low in the early stages of recovering renal function, and thus the full excretory potential of the CRRT devices should be exhausted.

• After termination of a CRRT cycle for the abovementioned reasons, anuria alone is not a sufficient criterion to immediately start the next cycle. In fact, CRRT is only restarted if hyperkalemia (> 5.5 mmol/l), hypervolemia (evidenced by congestive heart failure, relevant edema or impaired oxygenation) or profound uremia is present. There is no fixed threshold for the last criterion; CRRT is continued when patients with elevated serum urea levels have neurological symptoms that cannot be explained by other circumstances.

### Definition of "CRRT-free intervals" and their outcome

A "CRRT-free" interval as defined for this study had to meet two criteria: First, the CRRT cycle had to be actually terminated (CRRT device disassembled) and not simply temporarily interrupted, e.g. for diagnostic or therapeutic measures outside the ICU. Second, after termination of the CRRT cycle there was no compelling need to restart CRRT immediately, meaning that either of two outcomes i.e. restoration of an adequate urinary output or the resumption of CRRT were theoretically possible.

To assure that only CRRT-free periods as defined in the protocol were included, we only analyzed CRRT-free intervals that lasted 12 hours or longer. Using this cut-off value, we were able to exclude with a high degree of certainty temporary interruptions of CRRT and CRRT-free periods, in which it was obvious from the very beginning that it would be necessary to continue the treatment.

Restoration of an adequate renal function was assumed if the patients were discharged from the ICU and no further renal replacement therapy was implemented during the remaining hospital stay.

### Data processing

Using the electronic patient data management system "GISI" (Göttinger Informations-System für Intensivmedizin), we identified all patients in whom CRRT had been performed during the study period. A total of 448 CRRT-free periods matching the study definition were identified involving 222 patients. For each of these occasions, we extracted a standardized set of 29 parameters with an assumed influence on renal function. These parameters contained either general information (i.e. gender or age) or were related to the 12 hours immediately prior to or the first eight hours following the cessation of CRRT. The "Sequential Organ Failure Assessment"-score (SOFA score) was calculated at each cessation of CRRT to assess the severity of organ dysfunction. Tables 1 and 2 show the complete listing of all analyzed parameters; data were recorded anonymously in Microsoft Excel.

**Table 1:** Univariate analysis of continuous parameters within the evaluation group.

	Outcome of CRRT-free interval		p-value
	Recovery of kidney function N = 103	Resumption of CRRT N = 122	
General Data			
Age (years)	70 (IQR: 65-77)	74 (IQR: 61-78)	0.66^2^
SOFA score	8.3 (SD: 3.62)	9.2 (SD: 3.78)	0.042^1^
Number of CRRT cycles	1 (IQR: 1-1.75)	2 (IQR: 1-4)	< 0.0001^2^
			
Fluid balance (during the last 12 h of CRRT)			
Total balance (ml)	-315 (IQR: -807 to 214)	-336 (IQR: -1148 to 314)	0.74^2^
Urine output (ml/h)	33 (IQR: 14.2-54.2)	8 (IQR: 0-20)	< 0.0001^2^
Infused synthetic colloids (ml)	0 (IQR: 0-0)	0 (IQR: 0-0)	0.61^2^
			
Fluid balance (first 8 h after end of CRRT)			
Total balance (ml)	443 (IQR: 43-1133)	909 (IQR: 502-1361)	0.0003^2^
Urine output (ml/h)	66 (IQR: 29-122)	10 (IQR: 0-29)	< 0.0001^2^
Infused synthetic colloids (ml)	0 (IQR: 0-0)	0 (IQR: 0-0)	0.46^2^
			
Inotropes and vasoactive drugs (8 h-maximum after end of CRRT)			
Norepinephrine (μg/min)	0 (IQR: 0-0)	0 (IQR: 0-0)	0.28^2^
Epinephrine (μg/min)	0 (IQR: 0-0)	0 (IQR: 0-0)	0.35^2^
Dobutamine (μg/kg/min)	0 (IQR: 0-0)	0 (IQR: 0-0)	0.45^2^
			
Hemodynamics (Averaged 8 h after end of CRRT)			
Mean arterial pressure (mmHg)	77 (IQR: 71-84)	73 (IQR: 67-80)	0.013^2^
Central venous pressure (mmHg)	11.2 (IQR: 8.6-15.3)	12.8 (IQR: 10.0-16.1)	0.049^2^
Heart rate (1/min)	86 (IQR: 77-94)	89 (IQR: 81-96)	0.053^2^
			
Laboratory values (first value after end of CRRT)			
Na+ (mmol/l)	139.9 (SD: 3.1)	139.6 (SD: 3.0)	0.49^1^
K+ (mmol/l)	4.65 (IQR: 4.44-4.91)	4.70 (IQR: 4.40-4.90)	0.58^2^
Creatinine (mg/dl)	1.90 (IQR: 1.50-2.60)	2.05 (IQR: 1.40-2.70)	0.98^2^
Urea (mg/dl)	52.4 (SD: 17.7)	52.9 (SD: 19.6)	0.83^1^
pH	7.45 (IQR: 7.42-7.48)	7.44 (IQR: 7.40-7.47)	0.018^2^
Standard bicarbonate (mmol/l)	26.4 (IQR: 24.4-28.6)	26.0 (IQR: 24.2-27.7)	0.17^2^
Hemoglobin (mg/dl)	9.8 (IQR: 9.2-10.6)	9.4 (IQR: 8.7-10.4)	0.014^2^
			
Furosemide (first 8 h after end of CRRT)			
Total i.v. dose (mg)	50 (IQR: 0-100)	0 (IQR: 0-0)	< 0.0001^2^

Normally distributed data were analyzed with the t-test for independent samples ^(1)^, not-normally distributed data with the Mann-Whitney-test ^(2)^.

**Table 2:** Univariate analysis of categorical parameters within the evaluation group using χ^2^-test

	Outcome of CRRT-free interval		p-value
	Recovery of kidney function N = 103	Resumption of further CRRT N = 122	
Gender			0.37
Male	71 (68.9%)	76 (62.3%)	
Female	32(31.1%)	46 (37.7%)	
			
Chronic kidney disease			0.17
Yes (GFR < 60 ml/min/1,73 m^2^)	54 (52.4%)	76 (62.3%)	
No (GFR ≥ 60 ml/min/1,73 m^2^)	49 (47.6%)	46 (37.7%)	
			
Respiration after end of CRRT			0.13
Spontaneous breathing	61 (59.2%)	85 (69.7%)	
Mechanically ventilated	42 (40.8%)	37 (30.3%)	
			
Atrial fibrillation after end of CRRT			0.26
Yes	29 (28.2%)	44 (36.1%)	
No	74 (71.8%)	78 (63.9%)	
			
Diuretics other than Furosemide			< 0.0001
Yes	45 (43.7%)	15 (12.3%)	
No	58 (56.3%)	107 (87.7%)	
			
Drugs with potential negative effect on renal function			0.051
Yes	6 (5.8%)	18 (14.8%)	
No	97 (94.2%)	104 (85.2%)	

### Statistical methods

The 448 data sets of the 222 patients were divided into an evaluation group (the 225 data sets from the first 123 patients) and a validation group (the remaining 223 datasets from 99 patients). Depending on the clinical course, each CRRT-free interval was allocated to one of two outcome groups: "CRRT continued" if clinical conditions required resumption of renal replacement therapy, or "Recovery of kidney function" if patients were discharged from the ICU and no further CRRT was necessary.

In the evaluation group, all 27 continuous parameters were tested for normal distribution using the D'Agostino-Pearson test. Next, all parameters were tested univariately for having different distributions in the two outcome groups. For continuous variables with normal distribution we used Student's t-test for independent samples, and for those without normal distribution we used the Mann-Whitney rank-sum test. For all categorical values, we used a χ^2^-test. Afterwards, all parameters with different distributions in the outcome groups were subjected to logistic regression to assess their independent influence on the clinical endpoint "restoration of adequate renal function".

Based on the regression equation (resulting from logistic regression), a formula was developed to calculate the logit(p) value for each CRRT-free interval, which in turn is a measure of the probability of the desirable outcome (i.e. restoration of a sufficient kidney function) [11]. Accordingly, the logit(p) value was calculated for all 223 CRRT-free intervals of the validation group. ROC-analysis was performed to calculate sensitivity and specifity of the logit(p) value to identify patients with recovering kidney function.

For all tests, the level of statistical significance was set at p < 0.05. Calculations were performed with Medcalc^® ^software (Medcalc, Mariakerke, Belgium).

## Results

### Patients and general data

Between 2003 and 2006, 222 patients in the surgical intensive care unit were treated with CRRT for acute renal failure after cardiac surgery. One hundred forty-four of the patients were male, and 78 were female. The median age was 71 years (IQR: 66 - 77 years); median height 169 cm (IQR: 163 - 175 cm) and median weight 79 kg (IQR: 68 - 88 kg). The surgical procedures performed were coronary artery bypass graft (CABG, n = 94), valve surgery (n = 60), combined CABG and valve surgery (n = 39) and other cardiac surgery (n = 29). The median SOFA score was 8 with an IQR of 6 to 11.

Although the number of CRRT-cycles ranged from 1 to 14, the median was 1 with an interquartile range of 1 - 3 cycles. As only CRRT-free periods with a minimum duration of 12 hours were included into the study, their median duration was 23.6 hours (IQR: 14.6 - 45.4 hours).

### Univariate analysis

In the evaluation group, 11 of the 27 parameters showed statistically significant differences between the two outcome-groups: SOFA score, number of previous CRRT cycles, urinary output over the last 12 hours before cessation of CRRT, fluid balance and urinary output over the first 8 hours after cessation of CRRT, mean arterial and central venous pressures (both averaged over the first 8 hours after cessation of CRRT), arterial pH, hemoglobin concentration, total dose of furosemide and administration of any other diuretics during the first 8 hours after cessation of CRRT (tables 1 and 2).

### Multivariate analysis (logistic regression)

Multivariate analysis of these parameters showed an independent influence on the outcome of CRRT-free intervals for three variables: SOFA score, number of previous CRRT cycles and urinary output during the first 8 hours after cessation of CRRT (Table 3). The overall model fit statistic was assessed using the Likelihood Ratio Test which revealed a high goodness-of-fit (p < 0.0001).

**Table 3:** Results of logistic regression within the evaluation group (endpoint: Restoration of a sufficient renal function)

Variable	Regression coefficient	Odds ratio	Odds ratio 95% CI	p
SOFA-score	-0.174	0.840	0.751 - 0.939	0.0022
Number of CRRT cycles	-0.802	0.449	0.307 - 0.656	< 0.0001
Urinary output (ml/h)	0.026	1.026	1.017 - 1.036	< 0.0001
Constant	1.695			

Entering the regression coefficients into the regression equation, the following formula resulted to calculate the logit(p) value from the three clinical variables:

$$\text{logit}\left(\text{p}\right)=1.695-0.174*\text{SOFA}-0.802*\text{number of CRRT cycles}+0.026*\text{urinary output }\left(\text{ml}/\text{h}\right)$$

### ROC-Analysis (Figure 1)

**Figure 1:**
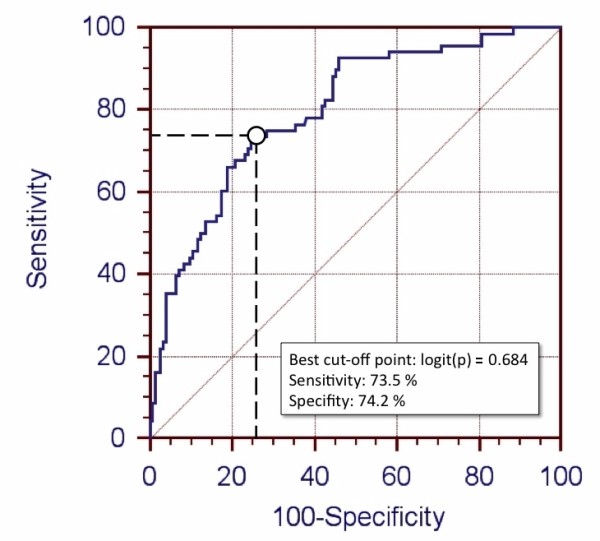
**Sensitivity and specificity of the calculated prognostic index for the prediction of recovery of renal function during CRRT-free periods**.

ROC-analysis of the calculated logit(p) values from the validation group revealed a sensitivity of 73.5% (95% CI: 61.4%-83.5%) and a specifity of 74.2% (95% CI: 66.6%-80.9%) for the identification of patients who would not require further CRRT. The optimal cut off point was a logit(p) value of 0.684, the area under the ROC-curve was 0.798 (95% CI: 0.740-0.894).

## Discussion

After termination of a CRRT-cycle, our score allows the prediction of whether another CRRT-cycle will be necessary or the kidneys will begin to function adequately. For the practical application two characteristics of the score are noteworthy: First, the index can be calculated timely, i.e. 8 hours after cessation of a CRRT cycle. Second, ROC-analysis gave an area under the curve of 0.798, which reflects a considerable discriminative power as measured by sensitivity and specifity.

Because renal function ultimately recovers in more than 90% of the patients [12], most renal replacement therapies are only temporary measures, and physicians must assess after each CRRT-cycle if another treatment cycle is necessary or not. In some ICUs, it is common practice to continue CRRT immediately after the end of a CRRT cycle (e.g. after the maximum operation time of the disposables has been reached), even if there is no mandatory indication for continuation. However, though many clinical trials have addressed this topic, no evidence exists at present showing that patients have any benefit from this kind of gapless therapy [13,14]. Moreover, even intermittent therapies have been proven to be as safe as CRRT for the vast majority of critically ill patients [3,4]. It is therefore also an absolutely reasonable approach to refrain from immediately continuing CRRT, if there are no clear indications for it, such as increasing serum potassium or volume overload. In addition to the medical and organizational impact, the decision to continue also has financial implications, as the disposable parts for a hemofiltration device alone cost about 250 € (roughly 330 USD) [15]. Against this background, a tool to predict the necessity of continuing CRRT may contribute to this decision in a helpful way.

When this study was planned, the most important prerequisite was a thorough assessment of the parameters with an assumed influence on renal function. An intensive search of the literature confirmed the influence of parameters such as arterial hypotension [16], hypovolemia [17], mechanical ventilation [18], the use of drugs with potentially harmful effects on kidney function [19] and the use of synthetic colloids [20]. All of these could be efficiently assessed from our electronic patient data management system. Additionally, we examined 16 other parameters with an assumed influence on kidney function for our statistical analysis, which also were obtained completely from the electronic patient data management system.

The comparison of our study design with those of Wu [9] and Uchino [10] is also of interest. With regard to the composition of the analyzed risk factors, all three trials integrated both general data (e.g. age, sex) and data which were variable at the end of each individual CRRT-cycle (such as fluid balance, hemodynamics etc.). However, Wu and coworkers did not take hemodynamic parameters into account, though it is known that hemodynamic instability is an important risk factor for acute kidney failure. On the whole, the resulting set of all analyzed risk factors in our trial is similar to the total of the risk factors chosen by Wu and Uchino.

It is interesting to note that serum concentrations of creatinine and urea after cessation of CRRT showed no significant differences between the two outcome groups in our study (creatinine: median 1.90 mg/dl and 2.05 mg/dl, p = 0.98, urea: mean 52.4 mg/dl and 52.9 mg/dl, p = 0.83). In addition to the serum concentrations, the increase of retention parameters can also be of interest for the assessment of kidney function. For instance, Ishani and coworkers found that the increase in serum creatinine might predict the progress of chronic kidney disease [21]. However, they analyzed the slope between baseline and peak serum creatinine level, which normally takes several days to reach. In contrast, our score was designed to predict the outcome of a CRRT-free interval soon after cessation of CRRT, wherefore it consists solely of data that can be retrieved within 8 hours after termination of CRRT. Due to the very slow rate of change of the two retention parameters, we intentionally did not analyze their increase after such a short time interval.

Of the parameters that showed different distributions in the two outcome groups, most of the differences were consistent with the expectations based on knowledge of renal injury. For example, mean arterial pressure, hemoglobin concentration and pH-value were higher in patients with recovering kidney function during CRRT-free intervals (table 1). The only exceptions were the total furosemide dose and the use of diuretics other than furosemide, which were both far higher in those patients who developed a satisfactory renal function during the CRRT-free intervals (furosemide dose in 8 hours: 82 mg vs. 18 mg, other diuretics: 45 out of 103 intervals vs. 15 out of 122 intervals). This is a remarkable finding because current data suggest that the potentially harmful effects of diuretics to an injured kidney can cancel out their benefit. Although a high urine flow might protect the tubular system from obstruction by cell detritus or sludge [22], diuretics can reduce medullar tonicity, which in turn might reduce renal blood flow [23]. In addition, diuretic-induced hypovolemia could aggravate prerenal causes of renal failure. The standard operating procedure in our ICU therefore prohibits the use of diuretics if the urinary output is below 0.3 ml/kg/h. Thus, the remarkably higher doses of diuretics in patients who did not need further CRRT are probably a consequence of recovering renal function, rather than its cause.

Basically, our results are concordant with the findings of Uchino and coworkers, who also investigated the possibility of predicting the outcome of CRRT-free periods [10]. In their trial, the amount of diuresis during CRRT also had a good discriminative power to predict the necessity of further renal replacement therapy. In contrast to their findings, serum creatinine was not significantly different between the two outcome groups in our trial. Moreover, Uchino only tested the discriminative power of two single parameters (urinary output and serum creatinine), but did not integrate them using an equation as we did. This simplifies the prediction, because no extensive calculations are required. However, the combination of several factors with an independent influence on the outcome may increase sensitivity and specifity of the prognosis [24]

A strongpoint of the present study is that all 27 parameters with an assumed influence on renal function were available in their entirety without any missing data. Moreover, hemodynamic parameters and fluid balances were recorded at one-minute intervals, which allowed for an analysis with a high degree of precision. In contrast, a primary weakness of the trial is its limitation to these 448 data sets. Obtaining a greater number of data sets would have required the analysis of patient data from a period significantly longer than three years or would have required a multicenter approach. This would have resulted in an increased inconsistency of treatment standards, and so the analysis of a 3-year interval was chosen as a suitable compromise between volume and homogeneity of the data sets. Due to the specialization of our ICU, only patients after cardiac surgery were included in the analysis, which is another limitation of the study.

## Conclusion

Only three of a total of 27 studied parameters showed an independent influence on the recovery of renal function after cessation of CRRT: SOFA score, number of previous CRRT cycles and urinary output after termination of CRRT. Nevertheless, a prognostic index based on these variables showed satisfactory power to predict the outcome of CRRT-free intervals in a separate validation group. To assess the general suitability of our results, validation in patients from other centers is required.

## Competing interests

The authors declare that they have no competing interests.

## Authors' contributions

DH developed the study design and drafted the manuscript. DG extracted all data from the patient data management system and prepared them for statistical analysis. OM made important contributions to the study design, especially to the composition of analysed risk factors. AB gave broad advices for statistical analysis and methods, and MQ critically revised all versions of the manuscript. All authors read and approved the final manuscript.
